# Epithelial requirement for in vitro proliferation and xenograft growth and metastasis of MDA-MB-468 human breast cancer cells: oncogenic rather than tumor-suppressive role of E-cadherin

**DOI:** 10.1186/s13058-017-0880-z

**Published:** 2017-07-27

**Authors:** H. J. Hugo, N. P. A. D. Gunasinghe, B. G. Hollier, T. Tanaka, T. Blick, A. Toh, P. Hill, C. Gilles, M. Waltham, E. W. Thompson

**Affiliations:** 1 Invasion and Metastasis Unit, St. Vincent’s Institute, Melbourne, VIC Australia; 2 Institute of Health and Biomedical Innovation, Queensland University of Technology, Brisbane, QLD Australia; 3 School of Biomedical Sciences, Queensland University of Technology, Brisbane, QLD Australia; 4 Translational Research Institute, Woolloongabba, QLD Australia; 5 Department of Surgery, University of Melbourne, St. Vincent’s Hospital, Melbourne, VIC Australia; 6 Department of Pathology, University of Melbourne, Melbourne, VIC Australia; 7 Interdisciplinary Cluster for Applied Genoproteomics (GIGA)-Cancer, Laboratory of Tumor and Development Biology, University of Liège, Liège, Belgium; 8 Institute of Health and Biomedical Innovation, Queensland University of Technology, Brisbane, Australia; 9 Australian Prostate Cancer Research Centre-Queensland, Brisbane, Australia

**Keywords:** Breast cancer, E-cadherin, Epithelial-to-mesenchymal transition, Epithelial-mesenchymal plasticity, Proliferation, Metastasis

## Abstract

**Background:**

Epithelial-to-mesenchymal transition (EMT) is associated with downregulated E-cadherin and frequently with decreased proliferation. Proliferation may be restored in secondary metastases by mesenchymal-to-epithelial transition (MET). We tested whether E-cadherin maintains epithelial proliferation in MDA-MB-468 breast cancer cells, facilitating metastatic colonization in severe combined immunodeficiency (SCID) mice.

**Methods:**

EMT/MET markers were assessed in xenograft tumors by immunohistochemistry. Stable E-cadherin manipulation was effected by transfection and verified by Western blotting, immunocytochemistry, and quantitative polymerase chain reaction (qPCR). Effects of E-cadherin manipulation on proliferation and chemomigration were assessed in vitro by performing sulforhodamine B assays and Transwell assays, respectively. Invasion was assessed by Matrigel outgrowth; growth in vivo was assessed in SCID mice; and EMT status was assessed by qPCR. Hypoxic response of E-cadherin knockdown cell lines was assessed by qPCR after hypoxic culture. Repeated measures analysis of variance (ANOVA), one- and two-way ANOVA with posttests, and paired Student’s *t* tests were performed to determine significance (*p* < 0.05).

**Results:**

EMT occurred at the necrotic interface of MDA-MB-468 xenografts in regions of hypoxia. Extratumoral deposits (vascular and lymphatic inclusions, local and axillary nodes, and lung metastases) strongly expressed E-cadherin. MDA-MB-468 cells overexpressing E-cadherin were more proliferative and less migratory in vitro, whereas E-cadherin knockdown (short hairpin CDH1 [shCDH1]) cells were more migratory and invasive, less proliferative, and took longer to form tumors. shCDH1-MDA-MB-468 xenografts did not contain the hypoxia-induced necrotic areas observed in wild-type (WT) and shSCR-MDA-MB-468 tumors, but they did not exhibit an impaired hypoxic response in vitro. Although vimentin expression was not stimulated by E-cadherin knockdown in 2D or 3D cultures, xenografts of these cells were globally vimentin-positive rather than exhibiting regional EMT, and they expressed higher *SNA1* than their in vitro counterparts. E-cadherin suppression caused a trend toward reduced lung metastasis, whereas E-cadherin overexpression resulted in the reverse trend, consistent with the increased proliferation rate and predominantly epithelial phenotype of MDA-MB-468 cells outside the primary xenograft. This was also originally observed in WT xenografts. Furthermore, we found that patients with breast cancer that expressed E-cadherin were more likely to have metastases.

**Conclusions:**

E-cadherin expression promotes growth of primary breast tumors and conceivably the formation of metastases, supporting a role for MET in metastasis. E-cadherin needs to be reevaluated as a tumor suppressor.

**Electronic supplementary material:**

The online version of this article (doi:10.1186/s13058-017-0880-z) contains supplementary material, which is available to authorized users.

## Background

In 2016 alone, 3073 people died as a result of breast cancer in Australia [1], with essentially all breast cancer deaths being due to metastasis [2, 3]. Accumulating evidence suggests that epithelial-mesenchymal plasticity (EMP), which is critical for the formation of new tissues during embryonic development, also facilitates metastasis from carcinomas, including breast cancer [4, 5]. In parallel to its role in normal mammary gland development [6–11], EMP in the mesenchymal direction (epithelial-to-mesenchymal transition [EMT]) is responsible for converting a fraction of noninvasive tumor cells from a carcinoma into stemlike cells with the ability to resist therapies, migrate, invade, intravasate, and survive in the systemic circulation [12, 13]. The reverse, mesenchymal-to-epithelial transition (MET), is thought to allow cells the cohesiveness to colonize new sites (reviewed in [4, 14–18]).

E-cadherin is a Ca^2+^-dependent transmembrane glycoprotein present in the epithelial cell membrane, maintaining intercellular adhesion through the formation of adherens junctions [19, 20]. E-cadherin plays a pivotal role in embryonic morphogenesis, mainly through governing early cellular differentiation pathways [21, 22]. Functional loss or downregulation of E-cadherin from epithelial cells is considered a hallmark of EMT [23, 24]. The first transcriptional suppressor of E-cadherin and inducer of EMT to be identified was the zinc finger transcription factor Snail family transcriptional repressor 1 *(SNAI1)* [24, 25]. Since then, other E-cadherin suppressors and EMT inducers, such as Snail family transcriptional repressor 2 *(SNAI2*, Slug), zinc finger E-box-binding homeobox 1 (*ZEB1*)/*TCF8*, *ZEB2* (*SIP-1*), Goosecoid, Twist-1, and Forkhead box protein C2 (*FOXC2*), have emerged [26–32]. All of these transcription regulators downregulate E-cadherin expression, potentially inducing EMT [33]. Given the striking similarities between cell translocation during embryonic morphogenesis and cancer metastasis [34, 35], E-cadherin has emerged as an important candidate regulator of the metastatic process in cancers of epithelial origin [36, 37].

Although EMT is a major mechanism contributing to tumor progression, unchecked cellular proliferation is the driver of tumor growth. Accumulating evidence suggests that EMT and cellular proliferation are inversely associated and that EMT attenuates cell proliferation in some [38, 39] but not all [40, 41] systems. Researchers in an early study on well-differentiated colorectal adenocarcinomas with lymph node (LN) metastasis reported loss of the proliferative marker Ki-67 in the cells along the invasive front in de-differentiated primary and secondary tumors, in contrast to the presence of high Ki-67 at the tumor center [42]. They observed diminished membranous E-cadherin and nuclear localized β-catenin in the Ki-67-negative cells at the invasive front, indicating attenuated proliferation in cells that have undergone EMT. This is reflected by the more recent finding that breast cancer stem cells located at the invasive front are primarily quiescent, whereas those in a more central location within the tumor are proliferative and retain the ability to transition between these states [43]. In turn, the antiproliferative drug cisplatin has been shown to induce EMT [44]. We have shown in breast cancer cells that suppression of proliferation by EMT is mediated at least in part through *ZEB1* repression of* MYB*, a gene important in driving proliferation of estrogen receptor (ER)-positive primary breast cancers [38].

MET is also critical in the developmental formation of new tissues as occurs in nephrogenesis [45], and it fuels the formation of metastatic tumors at the secondary site [4, 14, 18, 46–50]. Chao et al. [46] reported reexpression of E-cadherin in distant metastases arising in organs such as the liver, brain, and lung from primary breast tumors that were E-cadherin-low or E-cadherin-negative, and they suggested that the reexpression of E-cadherin in metastases was mediated by E-cadherin promoter demethylation influenced by the microenvironment of the metastatic site. Several other studies support the reexpression of E-cadherin at secondary sites [51–53]. This reexpression may reactivate cellular proliferation within the cells that emerge from the circulation at the secondary site, allowing them to form bulky metastases within the targeted niche organ, as reviewed elsewhere [14, 54, 55].

Although xenografts of MDA-MB-468 human breast cancer cells have been well described [56–58], relatively few studies have explored metastasis and EMP [59–61]. In the present study, we examined further the relationships between EMP status, proliferation, and metastasis through manipulation of E-cadherin in these cells.

## Methods

### Cell line and culture conditions

The MDA-MB-468 breast cancer cell line used in this study was originally obtained from the American Type Culture Collection (Manassas, VA, USA) by the Georgetown Lombardi Comprehensive Cancer Center (Washington, DC, USA) [62]. Cell cultures were routinely maintained in DMEM (Sigma-Aldrich, St. Louis, MO, USA) containing glucose (4500 mg/L), l-glutamine, and sodium pyruvate (110 mg/L) and supplemented with 10% FBS (SAFC Biosciences, Castle Hill, Australia). Cultures were maintained in antibiotic-free growth medium at 37 °C in a humidified incubator with O_2_ and CO_2_ levels set at 21% and 5%, respectively. For induction of hypoxia (HPX) in culture, the cells were passaged in DMEM supplemented with 10% FBS and incubated at 37 °C on an InvivO_2_ 400 workstation (Ruskinn Technology Ltd, Bridgend, UK) at 1% O_2_ and 5% CO_2_, and the cells were harvested at 48 h.

### Immunocytochemistry

MDA-MB-468 cells were plated in 96-well Terasaki plates (Nunc^TM^; Thermo Fisher Scientific, Roskilde, Denmark), and immunofluorescence was performed as previously described [38]. A volume of 12 μl per Terasaki well was used.

### Immunohistochemistry

Formalin-fixed, paraffin-embedded tissues were sectioned onto microscopic slides (SuperFrost® Plus; Menzel-Gläser, Braunschweig, Germany). Slides were then incubated in a humidified chamber with the primary antibody (antibodies used are listed in Table 2) and diluted in blocking buffer (PBS with 0.1% bovine serum albumin [BSA]) for 1 h at room temperature or at 4 °C overnight. The slides were next incubated with a biotinylated secondary antibody (raised against the immunoglobulin G [IgG] of the primary IgG species) for 1 h at room temperature, then incubated with HRP-conjugated streptavidin-biotin complex (Dako, Glostrup, Denmark) for 1 h at room temperature and stained with freshly prepared 3,3′-diaminobenzidine (DAB; Sigma-Aldrich). Slides were counterstained with hematoxylin for 5 minutes, mounted in dibutylphthalate polystyrene xylene (DPX), and coverslipped. The double immunohistochemistry (IHC) was performed using the BenchMark® ULTRA automated slide stainer (Ventana Medical Systems, Inc., Tucson, AZ, USA) in the Department of Pathology, St. Vincent’s Hospital, Melbourne, Australia. The chromogens used were ultraView® Universal Alkaline Phosphatase Fast Red (red color, vimentin) and ultraView® Universal DAB (brown color, E-cadherin) (both from Ventana Medical Systems, Inc.).

### Obtaining red-channel images

The color deconvolution plugin in ImageJ software (version 1.51j8; public domain program created by Wayne Rasband, National Institutes of Health, Bethesda, MD, USA) was used, set for Fast Red DAB, to obtain the red channel images in the E-cadherin/vimentin dual-stained images.

### Sulforhodamine B cell proliferation assay

The sulforhodamine B (SRB) colorimetric assay (Sigma-Aldrich) measures the amount of protein content, which is proportional to the number of cells [63]. The assays were performed in 96-well tissue culture plates containing 0.1 ml of culture medium per well and between 2500 and 20,000 cells per well. A series of plates were prepared with a desired number of cells per well and incubated at 37 °C in an incubator with O_2_ and CO_2_ set at 21% and 0.5%, respectively. The day of cell seeding was considered as day −2. One day after cell seeding (day −1), the normal culture medium was replaced with serum-free medium, followed by incubation for 24 h. Plates were fixed in 50% trichloroacetic acid (TCA), then 25 μl of prechilled (4 °C) 50% TCA was gently added to the growth medium in each well to give a final concentration of 10% TCA. The plates were then incubated at 4 °C for 1 h, followed by gentle washing with tap water. Washes were repeated five times for complete removal of TCA and growth medium and then air-dried overnight and stored at room temperature. At the end of the experiment, once all the plates had been TCA-fixed and air-dried, they were collectively stained for 30 minutes by adding 100 μl of freshly prepared 0.4% SRB (wt/vol) in 1% acetic acid per well. The plates were then washed five times with 1% acetic acid to completely remove protein-unbound stain. Plates were air-dried overnight at room temperature. SRB dye-bound protein appears bright pink in color. This was solubilized by adding 100 μl of 10 mM Tris base (pH 10.5) and incubating for 20–30 minutes on a gyratory shaker at room temperature. The optical density (OD) of absorbance at 540-nm wavelength was then measured in a microplate reader (POLARstar OPTIMA; BMG LABTECH, Ortenberg, Germany). The average of the OD values measured in the PBS-containing outer wells, which was generally about 0.04, was considered as the background OD. The results were analyzed using Prism version 5 software (GraphPad Software, La Jolla, CA, USA).

### Creation of modified cell lines

E-cadherin was exogenously expressed in MDA-MB-468 cells, referred to as 468-CDH1, by transfecting the cells with the plasmid hECD-pcDNA3 [64] using Lipofectamine^TM^ 2000 reagent (Invitrogen, Carlsbad, CA, USA) according to the manufacturer’s instructions. The cells were maintained in 600 μg/ml of G418 (geneticin; Invitrogen) in growth medium for 6–8 weeks to establish a stable, pooled cell line. Short hairpin RNA (shRNA)-expressing MDA-MB-468 cell pools, referred to as 468-shCDH1-A, -B, -C, or -D, were generated using the Lenti-X HTX packaging system (Clontech Laboratories, Mountain View, CA, USA). shRNA sequences (Table 1), along with a nonsilencing shRNA microRNA (miR) lentiviral control vector containing a scrambled sequence with no homology to any known mammalian genes (Open Biosystems, Lafayette, CO, USA), were encoded within a green fluorescent protein (GFP)-expressing lentiviral vector (pGIPZ; Open Biosystems). Successfully transduced cells were selected by cell sorting for GFP; thus, a transduced pool rather than individual clones was used in these studies.

**Table 1:** Short hairpin RNA used in this study

Cell line	Clone ID	Sequence
468-shCDH1-A	V2LHS_14834	CTGTTGGTGTCTTTATTAT
468-shCDH1-B	V2LHS_14838	GTCGTAATCACCACACTGA
468-shCDH1-C	V2LHS_14837	CCAACTGGCTGGAGATTAA
468-shCDH1-D	V2LHS_ 243170	GAGAGAGTTTCCCTACGTA

### Western blotting and quantitative real-time polymerase chain reaction experiments

These techniques were performed as previously described [38] to determine the degree of E-cadherin overexpression, depletion, or knockdown in the various modified cell lines. Antibodies used are detailed in Table 2. Quantitative real-time polymerase chain reaction (RT-qPCR) experiments were performed using gene-specific primers designed specially to detect either the exogenously expressed E-cadherin or the targeted sequences in shRNA-infected cells. Primer sequences are detailed in Table 3.

**Table 2:** Antibodies used in this study

	Species	Source	Catalogue number
Primary antibody			
Human E-cadherin	Rabbit	Abcam, Cambridge, UK	Ab15148
Human E-cadherin	Mouse	BD Biosciences, San Jose, CA, USA	610404
Human vimentin	Mouse	Dako, Glostrup, Denmark	M0725
α-Pan-actin	Mouse	Abcam, Cambridge, UK	Ab14128
Human mitochondrial	Mouse	Thermo Fisher Scientific, Waltham, MA, USA	MA5-12017
Mouse CD31	Rat	BD Pharmingen, San Diego, CA, USA	550274
Human HIF-1α	Mouse	BD Biosciences, San Jose, CA, USA	610958
Secondary antibody			
Antimouse biotin	Rabbit	Dako, Campbellfield, Australia	E0354
Antirabbit biotin	Swine	Dako, Campbellfield, Australia	E0431
Antirat biotin	Goat	Vector Laboratories, Burlingame, CA, USA	BA-9401
Antirabbit Alexa Fluor® 594	Donkey	Molecular Probes, Eugene, OR, USA	R37119
Antimouse Alexa Fluor® 488	Goat	Molecular Probes, Eugene, OR, USA	A11001
Tertiary label			
Streptavidin/HRP		Dako, Campbellfield, Australia	P0397

*HIF* Hypoxia-inducible factor

**Table 3:** Primers used in this study

Primer name		Nucleotide sequence
L32	RT sequence	CAGAAAACGTGCACATGAGCTGC
	Forward sequence (outer nested)	CAGGGTTCGTAGAAGATTCAAGGG
	Reverse sequence (outer nested)	CTTGGAGGAAACATTGTGAGCGATC
	Forward sequence (inner nested)	GATCTTGATGCCCAACATTGGTTATG
	Reverse sequence (inner nested)	GCACTTCCAGCTCCTTGACG
Carbonic Anhydrase 9 (for hypoxia validation)	Random priming used	
	Forward	CCTCAAGAACCCCAGAATAATGC
	Reverse	CCTCCATAGCGCCAATGACT
E-cadherin	RT sequence	GTCAGCCAGCTTCTTGAAGCGATT
	Forward sequence	GCCCTGCCAATCCCGATGAAA
	Reverse sequence	GGGGTCAGTATCAGCCGCT
Vimentin	RT sequence	CCGTGAGGTCAGGCTTGGAAA
	Forward sequence	GCTTCAGAGAGAGGAAGCCGAAAA
	Reverse sequence	CCGTGAGGTCAGGCTTGGAAA
Endogenous E-cadherin (CDH1-3′UTR)	RT sequence	GCACTTGGGGATTCTGGGCTTT
	Forward sequence	GTGCCTAAAGTGCTGCAGCCAAA
	Reverse sequence	GTACAAACCACGGATCTTGTGTCAGAAA
Exogenous E-cadherin (for 468-CDH1 construct)	RT sequence	GAAAGGACAGTGGGAGTGGCACTTT
	Forward sequence	CCTGAACTCCTCAGAGTCAGACAAA
	Reverse sequence	GTGGCACCTTCCAGGGTCAAGGAA
E-cadherin shRNA (for 468-shCDH1 constructs)	RT sequence	CCAGCTCAGCCCGAGTGGAAAT
	Forward sequence	CCTCCCATCAGCTGCCCAGAAAA
	Reverse sequence	CTCTGTCACCTTCAGCCATCCTGTTT
Snail 1	RT sequence	CGCAGACAGGCCAGCTCAGGAAT
	Forward sequence	CACATCCTTCTCACTGCCATGGAATT
	Reverse sequence	GCTGCCCTCCCTCCACAGAAAT
Estrogen Receptor 1	RT sequence	CCAGGGCCACGCTGGGAAATGAA
	Forward sequence	GTTCCAGTGGGCACTGTACTTGGATCTT
	Reverse sequence	CAGCTCCATGCCCCAGGGCTAAAT

*Abbreviations:* *RT* reverse transcription, *shRNA* Short hairpin RNA

### Transwell chemotaxis migration assay

The Transwell migration assay was performed using 24-well Transwell® permeable inserts containing polycarbonate membranes with 6.5-mm diameter, 8-μm pore size, and 0.3-cm^3^ bottom area (catalogue number 3422; Corning Life Sciences, Corning, NY, USA). MDA-MB-468 cells were resuspended at 1 × 10^6^ live cells/ml in FBS-free DMEM supplemented with 0.1% BSA. While the cell suspensions were prepared, 600 μl of the chemoattractant (DMEM supplemented with 10% FBS and 0.1% BSA) was dispensed into each well of the 24-well Transwell plates and warmed to 37 °C. The Transwell inserts were placed in the bottom wells containing prewarmed chemoattractant, and 1 × 10^5^ cells (100 μl from the cell suspension) were applied to each well. The Transwell plates were then incubated at 37 °C for 4.5 h, after which the growth medium in the inserts was removed and the membranes were washed twice in PBS. The membranes were then fixed in methanol for 1 minute and stained with Diff-Quik (Siemens, Bayswater, Australia) for 1 minute with eosin followed by 1 minute with buffered thiazole and washed with several changes of water to completely remove excess stain. The nonmigrated cells on the top side of the membranes were gently wiped off using wet cotton swabs. The membranes were left to air-dry overnight, carefully peeled off from the inserts, and placed onto microscopic slides with the migrated cells facing down, mounted in DPX mounting medium, and coverslipped. The number of migrated cells in five random high-power fields per membrane was counted using ImageJ software.

### Matrigel outgrowth assay (nubbin assay)

The Matrigel outgrowth assay was performed as described by Price and Thompson [65] using a 96-well plate setup. The desired number of cells (titration was done using 250, 500, and 1000 cells per well) were mixed in 5 μl of Matrigel and transferred into each well. The cell and Matrigel mixture was allowed to set by incubating at 37 °C for 15 minutes. Once the gel was set, 80 μl of DMEM supplemented with 10% FBS was carefully added to each well. The assay was then incubated at 37 °C for 1 week. The wells were photographed daily, and the morphological changes seen in the cells were assessed and recoded.

### Inoculation of severe combined immunodeficiency mice with E-cadherin-modified cell lines

MDA-MB-468 cells were grown in T175 flasks (Thermo Fisher Scientific, Australia) to approximately 70% confluence. Cells were trypsinized to a single-cell suspension, washed three times in PBS, and resuspended to 1.33 × 10^8^ viable cells in 1 ml of PBS. The cell suspension was kept on ice until used for inoculation. All of the experimental procedures pertaining to this section were performed according to the guidelines stipulated by the animal ethics committee (AEC) at St. Vincent’s Hospital, Melbourne (AEC protocol number 008/08). Severe combined immunodeficiency (SCID) mice (8–10 weeks old) were obtained from the animal resource center in Perth, Australia. Briefly, the mice were anesthetized with isoflurane, the lower abdominal region of the mouse was cleaned with 70% ethanol, and a vertical paramedial skin incision of about 1 cm was made in the lower right abdominal region overlying the mammary fat pad (MFP). The cell suspension (15 μl containing 2 × 10^6^ MDA-MB-468 cells) was injected into the MFP using a 50-μl Hamilton syringe. After inoculation of the cells, the incision was closed with two stainless steel wound clips, which were removed once the incision wounds were completely healed. The mice were monitored twice weekly throughout the experiment, and tumor dimensions and body weights were recorded. The volume of the tumor was estimated as width of the tumor squared multiplied by (length of the tumor divided by 2) and expressed in cubic millimeters. Mice were killed when the tumor volume reached 10% of the body weight. Tissues were harvested after mice were killed by exposing them to a high dose of CO_2_. The xenograft tumors were removed carefully, weighed, and photographed. One-half of the tumor was then fixed in formalin for histological analysis and IHC. The other half was chopped into small pieces and rapidly frozen in liquid nitrogen for RNA analysis. The lungs were fixed in formalin for histological analysis.

### Determining tumor/necrosis ratio

The tumor/necrosis ratio was assessed among the tumor groups by scanning the hematoxylin and eosin (H&E)-stained slides using Aperio ScanScope® slide scanner (Leica Biosystems, Buffalo Grove, IL, USA). The proportion of area representing viable tumor tissue and necrosis was then estimated with JMicroVision software.

### Analysis of human clinical data

All analyses of public and shared E-cadherin datasets were performed using the University of California Santa Cruz Xena program (http://xena.ucsc.edu/).

### Statistical analyses

Repeated measures analysis of variance (ANOVA) and two-way ANOVA with Bonferroni’s multiple comparisons posttest or Dunnett’s multiple comparisons posttest, one-way ANOVA, Student’s paired *t* tests, log-rank (Mantel-Cox) statistical tests, and Welch’s *t* tests were performed using Prism software. Where “*n*” is used (as in *n* = 3), this refers to the number of independent, biologic replicate experiments performed.

## Results

### MDA-MB-468 tumors exhibit regions of EMP associated with hypoxia

**Fig. 1:**
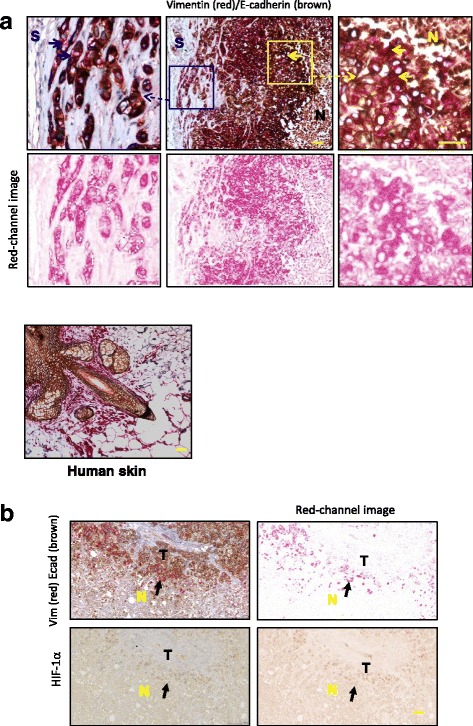
**a** Epithelial-to-mesenchymal transition (EMT) in MDA-MB-468 xenograft tumors. Vimentin-positive cells (*red*) indicating EMT were seen in regions along the tumor-stroma interface (*blue arrows*) and more continuously along the tumor-necrosis border (*yellow arrows*). The vimentin-positive cells at the invasion front were arranged in thin rows of individual cells interspersed between stromal (*S*) connective tissue (*blue arrows*). Vimentin-positive cells along the necrosis (*N*) front were also arranged in a thin border (yellow arrows). A *red-colored* deconvoluted image is shown to enable discernment of vimentin immunohistochemical staining. E-cadherin/vimentin-positive control: human skin. Scale bars represent 50 μm. **b** Evidence of epithelial-mesenchymal plasticity in MDA-MB-468 cell xenografts in regions associated with hypoxia. Images at *right* are red-channel images (derived from image using the ImageJ Colour Deconvolution plugin) of images at *left* to more clearly illustrate features. Area of necrosis is denoted with *yellow N*, tumor is denoted with *black T*. Hypoxia-inducible factor (HIF)-1α-positive cells were seen along the tumor-necrosis border (*black arrow*), and the area was also positive for vimentin (scale bar = 50 μM). For another example, *see* Additional file 1: Figure S1

Consistent with previous results [66], the differential EMP status in these xenograft tumors was demonstrable by double IHC of E-cadherin (Fig. 1a, *brown*) and vimentin (Fig. 1a, *red*). Vimentin-positive cells (*red color*) indicating EMT were clearly seen in two distinct areas of the tumor: at regions of the tumor-stroma interface (*blue arrows*) and along the tumor-necrosis border (*yellow arrows*). Cells between these two layers were mainly vimentin-negative and E-cadherin-positive (*brown color*). Some of the vimentin-positive cells in the tumor-stroma interface were also positive for E-cadherin, indicating a hybrid phenotype. The vimentin-positive cells at the invasion front were arranged in thin rows of individual cells interspersed between stromal (S) connective tissue (*blue arrow*), typical of the invasive lobular carcinoma that lacks E-cadherin [67]. Vimentin-positive cells along the necrosis (N) front were also arranged in a thin border (*yellow arrow*). Human skin was used as the positive control for both E-cadherin and vimentin staining (Fig. 1a).

We hypothesized that the EMP seen along the tumor-necrosis border could be a consequence of HPX, given recent reports of HPX-induced EMT in MDA-MB-468 cells [68, 69] in addition to our own in vitro study [70]. To assess HPX in the xenograft tumors, IHC staining of the HPX marker hypoxia-inducible factor-1α (HIF-1α) was performed. HIF-1α-positive cells (*black arrow*) were distributed along the tumor (*T*)-necrosis (*N*) border only, corresponding to the region containing the vimentin-positive cells (Fig. 1b, *black arrow*), as also seen in islands of tissue surrounding vessels within the necrotic region (Additional file 1: Figure S1a). These combined results associate the necrotic interface EMT with HPX and suggest an HPX-independent EMT induction mechanism inducing vimentin at the tumor-stroma border.

### Metastases of MDA-MB-468 xenograft primary tumors exhibit evidence of epithelial differentiation, consistent with MET

**Fig. 2:**
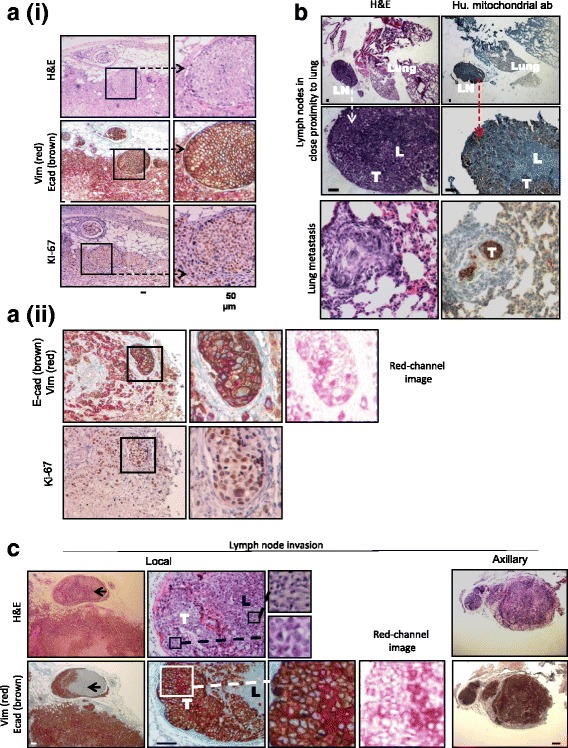
Pronounced expression of E-cadherin was observed in MDA-MB-468 xenograft metastases. **a** (*i*) and (*ii*) Similarity of features of MDA-MB-468 xenograft tumors. Red-channel image is shown for simplicity of vimentin staining. **b** Metastatic tumor cells in lymph nodes (LNs) and lungs. Positive staining for an antihuman mitochondrial antibody confirmed that tumor cells were of human origin (*T*), whereas mouse lymphocytes (*L*) were not stained. Tumor cells in the lung demonstrated E-cadherin expression but not vimentin. **c** LN invasion. *Black arrows* indicate invasion of E-cadherin and vimentin-expressing tumor cells to the adjacent LNs (*T*). Red-channel image is shown for clarity of vimentin staining. Tumor cells in axillary nodes stained homogeneously for E-cadherin. Lymphoid tissue (*L*) did not stain for either E-cadherin or vimentin, confirming its murine origin. All scale bars = 50 μm, except for axillary LN images, where scale bar represents 100 μm. *H&E* Hematoxylin and eosin

An interesting histological feature observed in the MDA-MB-468 tumors was the existence of carcinoma in situ-like areas (Fig. 2a). These spherical tumor masses were surrounded by connective tissue fibers, presenting as encapsulated tumor. They were generally located toward the periphery of the tumors and were composed of cohesive tumor cells that strongly expressed E-cadherin, in contrast to the neighboring vimentin-positive cells at the invasive front of the tumor. As shown in Fig. 2a(i) and (ii), the pattern of Ki-67 staining in the MDA-MB-468 tumors did not correlate with either vimentin or E-cadherin staining in the tumor proper or in the zones of EMT, and it was expressed in approximately 90% of all cells in the growing tumor.

As shown in Fig. 2b, metastatic tumor cells were seen in some LNs within close proximity of the lungs and within the lungs themselves, with positive staining with a human-specific mitochondrial antibody confirming that these cell clusters were of human origin. Lung micrometastases were seen across all the groups, with a marked trend toward E-cadherin-expressing groups (Table 4).

**Table 4:** Incidence of lung metastases across the tumor groups

Group	Number observed	With lung metastasis	Percentage of metastasis
468-shSCR	10	7	70
468-shCDH1-B	10	3	30
468-shCDH1-D	10	5	50
468-pcDNA3	9	5	55.6
468-CDH1	10	7	70

Fisher’s exact test was used to determine statistical significance

MDA-MB-468 tumor cells were detected in LNs associated with the lungs and as micrometastases within the lungs themselves (Fig. 2c). Confirmation of the presence of cells of human origin was obtained by a human-specific, antimitochondrial antibody (*T*) with mouse unstained (*L*) (Fig. 2c, *inset*). Most of the cells demonstrated E-cadherin expression; however, none were positive for vimentin, indicating an epithelial phenotype. Both local and distant LN (axillary) metastases were observed in a subset of mice (*n* = 4) with MDA-MB-468 xenograft tumors (Fig. 2c). The tumor cells that invaded into the local LNs generally displayed a heterogeneous expression pattern of E-cadherin and vimentin, as shown in the enlarged image, an indication that these cells displayed a spectrum of EMP, although epithelial cells with strong E-cadherin expression were the most abundant. In contrast, tumor cells in the axillary nodes stained homogeneously for E-cadherin. These results support a requirement for epithelial phenotype for secondary colonization by metastasized tumor cells and support the possibility of MET.

### Creation and validation of E-cadherin-modified MDA-MB-468 cell lines

Given that EMP was observable in MDA-MB-468 cells grown in vivo in xenografts (Fig. 1) and that E-cadherin was a strong feature of secondary deposits (Fig. 2), we sought to enforce either stable epithelial or mesenchymal status in the MDA-MB-468 cell system by directly manipulating E-cadherin expression positively or negatively, respectively, and to examine MDA-MB-468 cell behavior in vivo. Manipulation of E-cadherin has been used effectively for EMP studies in HMLE human epithelial mammary cells [71], human head and neck squamous carcinoma cells [72], and dog kidney and mouse mammary carcinoma cells [73].

**Fig. 3:**
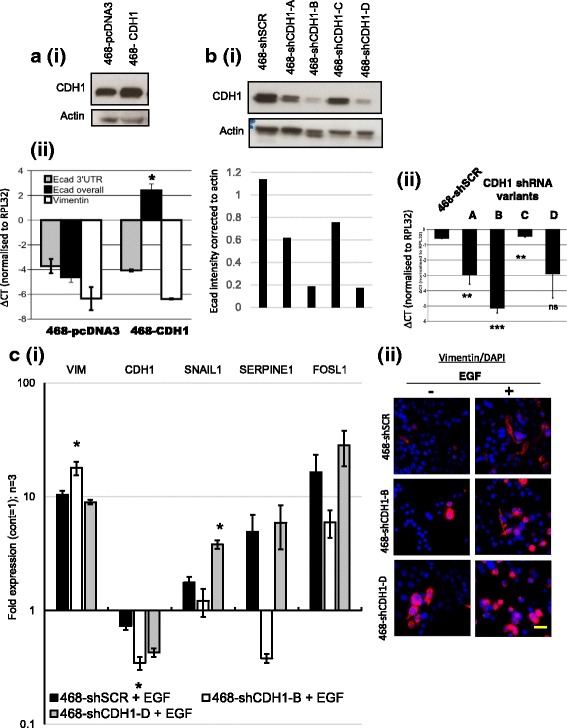
Validation and characterization of E-cadherin-manipulated MDA-MB-468 cell lines. E-cadherin-overexpressing (**a**) and short hairpin RNA (shRNA)-transduced (**b**) MDA-MB-468 cells assessed by (*i*) Western blotting and (*ii*) quantitative polymerase chain reaction (qPCR) (error bars represent SEM in three independent experiments). Statistical significance was determined by two-tailed, paired *t* test; *** *p* < 0.0001, ** *p* < 0.01 and * *p* < 0.05. **c** (*i*) Treatment of 468-shCDH1 cells with epidermal growth factor (EGF) to assess their ability to undergo epithelial-to-mesenchymal transition (EMT) by qPCR for various EMT genes. Fold expression (from the control) is shown, where control = 1 and *y*-axis is on a logarithmic scale. Error bars represent SEM derived from three independent experiments. (*ii*) Matching vimentin immunofluorescence (combined with nuclear 4′,6-diamidino-2-phenylindole [DAPI] stain) of the cells at day 5 time point (scale bar represents 50 μm). *ΔCT* Cycle threshold change, *UTR* Untranslated region

MDA-MB-468 cells were transfected with either full-length human E-cadherin (468-CDH1) or four shRNA variants (468-shCDH1-A–468-shCDH1-D) and a “scrambled” nontargeting hairpin control (468-shSCR) against CDH1, as described in the “Methods” section above. Each transfectant/transductant was characterized by Western blotting and qPCR (Fig. 3a and b).

Western blotting (Fig. 3a, *i*) confirmed an elevated E-cadherin protein level in 468-CDH1 cells compared with 468-pcDNA3 cells. Gene-specific qPCR analysis (Fig. 3a, *ii*) was performed using primers designed to detect endogenous versus exogenous forms of E-cadherin (*see* Table 3 for primer sequences). The collective expression of both exogenous and endogenous E-cadherin was detected using a more general primer set (i.e., “E-cad overall” as labeled in Fig. 3a, *ii*). The endogenous E-cadherin messenger RNA (mRNA) was assessed using a primer pair targeting the 3′ untranslated region of the *CDH1* gene. Both the cell types examined in Fig. 3a(iii) expressed endogenous E-cadherin; however, the 468-CDH1 cells also expressed exogenous E-cadherin, which contributed to the E-cadherin measured in the overall primer set, confirming the successful transfection of full-length E-cadherin into MDA-MB-468 cells. Approximately 130-fold induction (2^−ΔΔCt^) was observed in the expression of overall E-cadherin message, which was statistically significant in 468-CDH1 cells compared with 468-pcDNA3 cells, with no significant change in endogenous E-cadherin expression. Vimentin expression in 468-CDH1 cells remained unchanged from the basal level, despite their forced epithelial status (Fig. 3a, *ii*).

Characterization of shRNA variants compared with the scrambled control by Western blotting showed that the 468-shCDH1-B and 468-shCDH1-D cells expressed the least E-cadherin protein, and the least was seen in 468-shCDH1-C cells (Fig. 3b, *i*). A comparable pattern was also seen by qPCR (Fig. 3b, *ii*). As shown in Fig. 3c, the best E-cadherin knockdown confirmed by qPCR (468-shCDH1-B) exhibited further upregulated vimentin expression and E-cadherin repression above that of the SCR short hairpin control when the cell lines were treated with epidermal growth factor (EGF) for 5 days. The EMT-related genes serpin family E member 1 (*SERPINE1*) and *FOSL1* selected for analysis were based upon a previously published gene set consistently upregulated in our hands in MDA-MB-468 cells that underwent EMT after treatment with either EGF or HPX [70]. Snail1 was selected because it was shown to be upregulated by EGF in this previous study. All EMT genes were upregulated by EGF in all cell lines tested, except for *SERPINE1*, which was downregulated in 468-shCDH1-B cells. Immunofluorescence for vimentin at this 5-day treatment time point (Fig. 3c, *ii*) reflected the induction by EGF observed by qPCR.

### Forced E-cadherin expression blocked cellular invasion, whereas its knockdown stimulated invasion and reduced proliferative growth

**Fig. 4:**
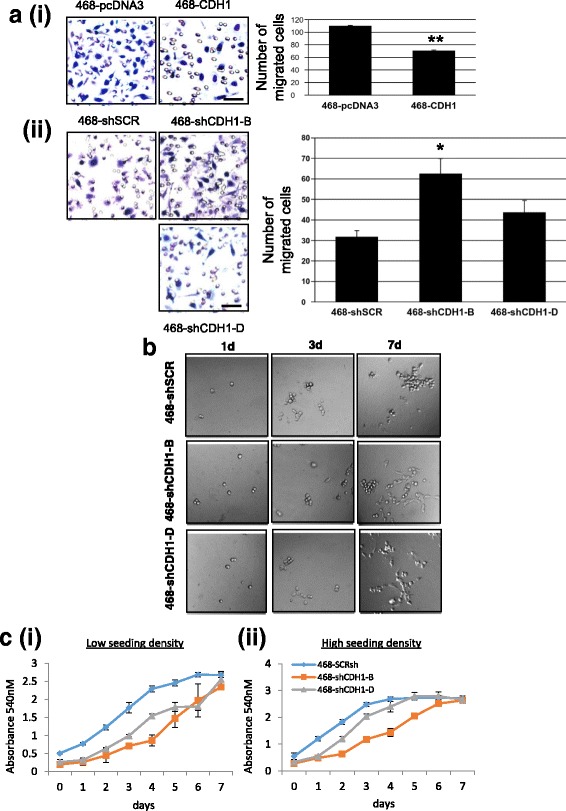
**a** Transwell chemotaxis migration assay (*n* = 3). Error bars represent SEM, and statistical significance was determined by two-tailed paired *t* test; ** *p* < 0.0025, * *p* < 0.01. Original magnification × 200, scale bar = 50 μm. Images shown are representative only of the quantification shown on *right*; quantification was determined from the entire cell layer. **b** Matrigel outgrowth (nubbin) assay to assess invasive potential. **c** Sulforhodamine B assay (*n* = 3). Error bars represent SEM, and statistical significance was determined by two-way analysis of variance with Bonferroni’s posttest; * *p* < 0.001. (i) Low seeding density: 2500 cells/well; (ii) high seeding density: 5000 cells/well seeding density. *ns* Not statistically significant

We examined the invasive potential and proliferative rate of the various E-cadherin-modified cells in culture (Fig. 4).

Each cell line was assessed in the Transwell chemomigration assay. The number of migrated 468-CDH1 cells was significantly lower than the control 468-pcDNA3 control cells, indicating that overexpression of E-cadherin in MDA-MB-468 cells significantly reduced their potential to migrate as single cells (Fig. 4a, *i*). Compared with the 468-shSCR cells, the number of migrated 468-shCDH1-B cells was significantly higher (Fig. 4a, *ii*). Similarly, a higher rate of cell migration was apparent in 468-shCDH1-D compared with the vector control, although this was not statistically significant. The results highlight that the functional knockdown of E-cadherin achieved by shRNA in MDA-MB-468 cells led to an increase in migratory capacity. Similarly, the 468-shCDH1-B and 468-shCDH1-D cells were more invasive, as shown by greater Matrigel outgrowth (Fig. 4b). In this assay, cells were evenly dispersed in a nubbin of Matrigel that was formed on a Matrigel-coated well in a 96-well setup, topped with FBS-supplemented DMEM and incubated at 37 °C. By the seventh day of the assay, a portion of 468-CDH1shRNA-B and 468-CDH1shRNA-D cells had invaded through the 3D culture coating to the floor of the well, where they displayed an elongated phenotype resembling mesenchymal cells. The noninvaded cells remained trapped in the Matrigel and formed a spheroidal cluster of cells similar to the cell clusters formed by 468-SCRshRNA cells. These results show that knockdown of E-cadherin in MDA-MB-468 cells enhanced their invasive potential.

The SRB assay was performed to compare the rates of cell proliferation. To determine if density affected this, the cells were seeded at two different cell densities (2500 and 5000 cells per well; *see* Fig. 4c, *i* and *ii*, respectively). No significant difference was observed between 468-pcDNA3 and 468-CDH1 cell lines with respect to proliferative rate (data not shown); however, 468-shCDH1-B and 468-shCDH1-D cells proliferated at noticeably lower rates than the 468-shSCR control when plated at low density (Fig. 4c, *i*). At twice the seeding cell density, an initial lag was observed in the proliferative rate of 468-shCDH1-B and 468-shCDH1-D cells, which gradually disappeared by the sixth day (eighth day after cell seeding). The results reveal that E-cadherin silencing has an inhibitory effect on cell proliferation, which was more prominent under sparse culture conditions.

### E-cadherin-manipulated tumors grew slower than their respective controls

**Fig. 5:**
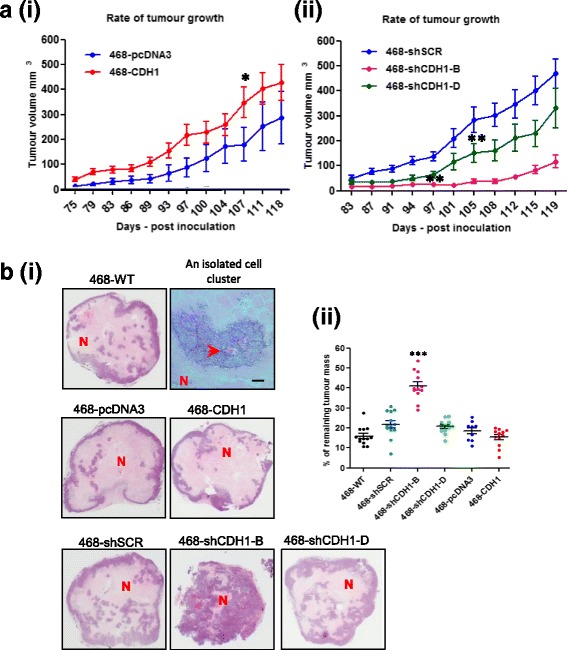
**a** Rate of xenograft tumor growth. Individual tumor volumes from each mouse from each group were averaged and plotted against the number days postinoculation. Statistical significance was determined by two-way analysis of variance (ANOVA) with Sidak’s posttest; ** *p* < 0.001, * *p* < 0.05. (*i*) E-cadherin overexpression group. (*ii*) E-cadherin knockdown group. **b** The tumor/necrosis ratio. (*i*) Hematoxylin and eosin staining of xenograft tumors, low magnification. *N* Necrotic area. Blood vessels are indicated by *red arrowhead*. Scale bar = 50 μm. (*ii*) Percentage of viable tumor tissue/remaining tumor mass. Statistical significance was determined by one-way ANOVA; *** *p* < 0.001

We tested the primary tumor growth and metastatic competence of the E-cadherin-manipulated cell lines by orthotopically inoculating them into the MFPs of SCID mice and subsequently analyzing the primary xenograft tumor growth and extent of lung metastases. Details of the cell groups used in the in vivo experiments are listed in Table 5. No significant difference was detected between the 468-shSCR and 468-pcDNA3 groups from days 80 to 119 (data not shown). As shown in Fig. 5a(*i*), the 468-CDH1 xenografts appeared larger than the 468-pcDNA3 ones at all time points and were significantly larger on day 107, as determined by two-way ANOVA with Bonferroni’s posttest.

**Table 5:** Summary of the in vivo experiments

Group	Number of mice inoculated	Tumors	Died early	Tissue harvest
468-WT	13	13	0	13
468-shSCR	13	13	0	13
468-shCDH1-B	13	13	0	13
468-shCDH1-D	13	13	01	12
468-pcDNA3	12	09	0	12
468-CDH1	12	12	0	12

*sh* Short hairpin, *SCR* Scrambled, *WT* Wild type

The E-cadherin-modified MDA-MB-468 cell lines were orthotopically inoculated to the right mammary fat pads of severe combined immunodeficiency mice (*n* = 12–13 per group). The groups inoculated with wild-type MDA-MB-468 (468-WT), 468-shSCR, 468-shCDH1-B, 468-shCDH1-D, and 468-CDH1 developed tumors in all mice. In the 468-pcDNA3 group, 9 of 12 mice developed tumors. One mouse from the 468-shCDH1-D group died before harvest as a result of non-tumor-related causes

The rate of tumor growth was more markedly different among the 468-shSCR, 468-shCDH1-B, and 468-shCDH1-D groups (Fig. 5a, *ii*). Although no obvious changes were observed between 468-shSCR and 468-shCDH1-B at the earlier time points, the tumor volume was significantly lower in 468-shCDH1-B than in the vector control 468-shSCR from 97 days onward postinoculation. A similar trend was also detected between the 468-shSCR and 468-CDH1shRNA-D groups, although the difference in tumor growth was not seen until 105 days postinoculation. No difference was seen between 468-shCDH1-B and 468-shCDH1-D for up to 136 days postinoculation, after which 468-shCDH1-D tumors demonstrated a significantly higher tumor growth rate than 468-shCDH1-B (Fig. 5a, *ii*; Additional file 2: Figure S2a). The results revealed that the tumor growth rate in E-cadherin-knockdown groups was significantly slower than their vector control, and this appeared to be dose-dependent because 468-shCDH1-B had a more complete knockdown than 468-shCDH1-D. Despite these marked changes in tumor growth, no difference was observed in Ki-67 staining in tumors from the E-cadherin-modified cell lines (Additional file 3: Figure S3).

### E-cadherin-knockdown tumors are less necrotic

Standard histopathological assessment of the xenografts was performed by H&E staining. The tumors from all groups except 468-shCDH1-B were composed of a central necrotic area surrounded by a thin rim of viable tumor tissue (Fig. 5b, *i*). A common feature detected in these tumors was the presence of small islands of viable cells surrounding a central blood vessel, embedded within the central necrotic region. Tumors from the 468-shCDH1-B group, regardless of tumor size, were composed mainly of viable tumor tissue with only very small areas of necrotic foci (Fig. 5b, *i*). The percentage of necrosis exceeded that of viable tumor tissue across all groups except 468-shCDH1-B, where the amount of viable tumor tissue was considerably higher than the extent of necrosis (Fig. 5b, *ii*). The proportion of viable tumor tissue was significantly higher in 468-shCDH1-B tumors than in any other group.

### Tumor microvasculature is more developed in 468-shCDH1-B xenografts

**Fig. 6:**
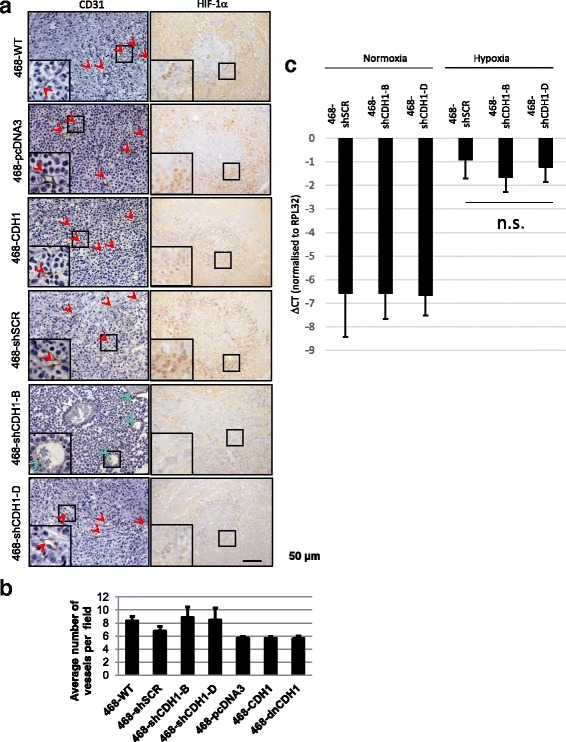
**a** Immunohistochemistry for CD31 (*left panel*), with smaller vessels indicated by *red arrowheads* and larger vessels indicated by *green arrowheads*. **b** Plot of the average number of CD31-stained blood vessels in five random microscopic fields (original magnification × 200) per tumor. Statistical significance was assessed by one-way analysis of variance (no difference across groups). **c**. Quantitative polymerase chain reaction data for hypoxic indicator gene carbonic anhydrase 9 (*CAIX*) (*n* = 3). Error bars represent SE. *ΔCT* Cycle threshold change, *SCR* Scrambled, *WT* Wild type

We investigated whether differences in necrotic area between the various tumor types (Fig. 5b) was due to altered vasculature using IHC staining of the vascular endothelial marker CD31/platelet endothelial cell adhesion molecule-1 (Fig. 6a, *left panel*). In 468-shCDH1-B tumors, the majority of blood vessels were larger with well-organized architecture, as compared with the less substantial microvasculature observed in the tumors across all other groups, whereas no statistically significant change was detected in the number of blood vessels across the tumor groups (Fig. 6b). All tumor groups except 468-shCDH1-B displayed positive HIF-1α staining (Fig. 6a, *right panel*), consistent with HPX occurring within these tumors. Where seen, these hypoxic regions correlated with vimentin positivity (data not shown), as was originally observed for WT MDA-MB-468 tumors (Fig. 1a).

E-cadherin knockdown in human breast cancer cell line xenografts has been shown to decrease tumor growth due to impaired HIF-1α expression and subsequent ability to metabolize glycogen as an energy source [74]. To determine whether the 468-shCDH1-B tumors did not express HIF-1α because of E-cadherin knockdown or whether the cells simply did not experience HPX in vivo, we cultured these cells in hypoxic conditions in vitro and assessed HIF-1α expression. As shown in Fig. 6c, 468-shCDH1-B and 468-shCDH1-D cells showed induction of the HIF-1α gene-regulated gene carbonic anhydrase 9 (*CAIX*) under HPX to a level similar to that of the SCR control, indicating that the hypoxic response was still intact, at least in vitro.

### EMT in xenograft tumors from E-cadherin-manipulated MDA-MB-468 cells

**Fig. 7:**
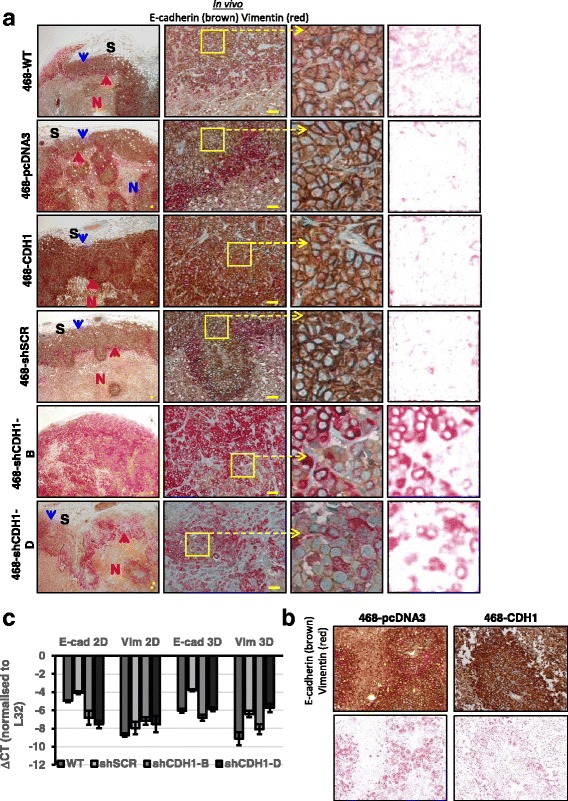
**a** Epithelial-mesenchymal plasticity status in vivo as xenografts in mice. E-cadherin was assessed by double-immunohistochemistry (IHC) of E-cadherin (*brown*) and vimentin (*red*). Vimentin-positive cells at tumor-stroma interface are indicated by *blue arrowheads*, and at tumor-necrosis border they are indicated by *red arrowheads*. Red-channel images are shown for vimentin IHC clarity. All scale bars = 50 μm. **b** Enlargement of 468-pcDNA3 and 468-CDH1 tumors showing differences in vimentin-E-cadherin staining. **c** Quantitative polymerase chain reaction expression of the same modified cells grown in 2D versus 3D. *ΔCT* Cycle threshold change, *SCR* Scrambled, *WT* Wild type

As seen in WT tumors (Fig 1a), in all tumors except 468-shCDH1-B and to some extent 468-shCDH1-D, vimentin-positive cells were observed at the invasion front along the tumor-stroma border and also at the tumor-necrosis border, indicative of EMP (Fig. 7a). Generally, as seen in the MDA-MB-468 WT tumors, the cells at the invasive front of the various xenograft tumors appeared to be arranged in thin rows in “Indian file” formation, interspersed among the stromal connective tissue. In addition, some individual cells were separated from the main tumor mass and located at the extreme periphery of the invasive front (indicated by *blue arrow* in 468-WT in vivo vimentin-E-cadherin low-power image in Fig. 7a). The majority of the cells at the invasive front expressed vimentin, indicating EMT in this area, whereas some of the cells coexpressed both vimentin and E-cadherin, highlighting the existence of a hybrid cell state able to respond quickly to external stimuli [75]. However, none of the cells at the invasive front demonstrated HIF-1α positivity, indicating a lack of HPX in this region (data not shown, but Fig. 1a is representative of these results). These observations support the existence of EMP at the invasive front, which is not a result of HPX.

In contrast, E-cadherin was expressed strongly in epithelial cells located in the tumor proper, in between the vimentin-positive borders, in 468-WT, 468-shSCR, 468-pcDNA3, and 468-CDH1 tumors (Fig. 7a). Although the E-cadherin was overexpressed in 468-CDH1 cells in vitro (mRNA) (Fig. 3s, *ii*), no visible difference was seen in the intensity of E-cadherin staining between 468-CDH1 and the vector control 468-pcDNA3 tumors; however, the ability to distinguish the two colors as separate entities (i.e., E-cadherin and vimentin) in the 468-CDH1 tumors did appear somewhat compromised (Fig. 7b). Vimentin induction in these tumors was not blocked, however, which was unexpected. A few areas of E-cadherin-expressing cells were seen in both 468-shCDH1-B and 468-shCDH1-D tumors; however, this appeared very weak compared with the 468-shSCR staining. Conversely, vimentin-expressing cells in the 468-CDH1 tumors were prominent at the tumor-stroma border, with one vimentin-positive cell being visible at high power (468-CDH1 high-power image shown in Fig. 7a, *green arrow*).

### E-cadherin-knockdown cells displayed EMT in vivo

In 468-shCDH1-B and 468-shCDH1-D tumors, the majority of the cells showed strong vimentin staining, indicating widespread EMT in these tumors (Fig. 7a). This is in contrast to the in vitro findings with these cells, where vimentin mRNA was not induced constitutively after E-cadherin reduction (Fig. 3c, comparing no EGF controls). To determine whether global induction of vimentin in these E-cadherin-knockdown tumors was due to the 3D state in vivo, we cultured each of the control and sh-CDH1 cells in 3D Matrigel compared with 2D monolayer cultures. We confirmed the lack of vimentin mRNA induction seen in 2D (Fig. 3) and found no further stimulation when the cells were cultured in the 3D environment (Fig. 7c).

**Fig. 8:**
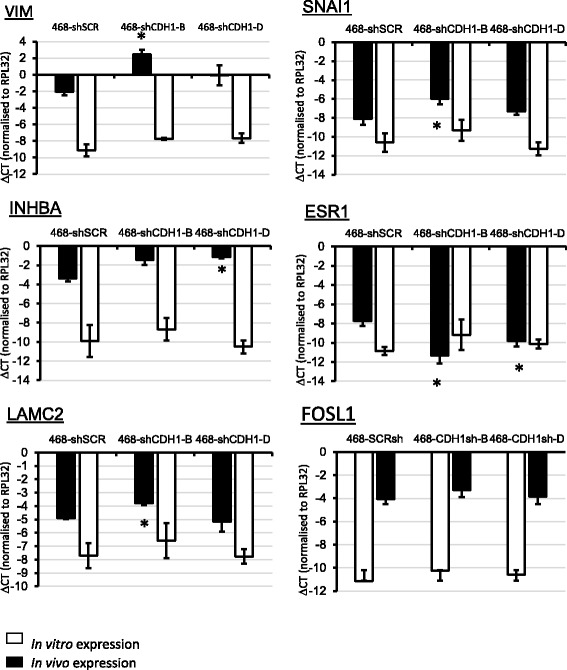
Quantitative polymerase chain reaction gene expression analyses of 468-shCDH cell xenografts (in vivo) versus plated in 2D culture (in vitro). Results shown are average expression of RNA extracted from three tumors or from three biological replicates of cells plated in 2D. Error bars represent SE. Significance was set at *p* < 0.05 and is indicated by asterisk and determined by Student’s paired *t* test (compared with 468-shSCR control). *ΔCT* Cycle threshold change, *SCR* Scrambled, *WT* Wild type, *ESR1* Estrogen receptor alpha, *INHBA* Inhibin, beta A, *SNAI1* Snail family transcriptional repressor 1

The increased in vivo expression of vimentin protein by IHC in 468-shCDH1-B tumors corresponded with upregulated expression of vimentin in matching xenograft tumor RNA (Fig. 8). *SNA1* was also significantly upregulated in these tumors. This correlated with a significant downregulation of the estrogen receptor alpha (*ESR1*), a known Snail1 transcriptional target for repression [76]. *SNA1* was variably expressed in a pattern similar to that observed for vimentin, where it was induced in vivo in the E-cad-knockdown tumors but not in vitro, and *ESR1* followed suit (Fig. 8). Inhibin, beta A (*INHBA*) and *LAMC2* were also upregulated in the E-cadherin-knockdown 468 lines grown in vivo, whereas this was not seen when the cells were plated in 2D culture, and *FOSL1* was more highly expressed in vivo (Fig. 8).

### E-cadherin was expressed in tumor emboli located within local vasculature, regardless of E-cadherin manipulation status

**Fig. 9:**
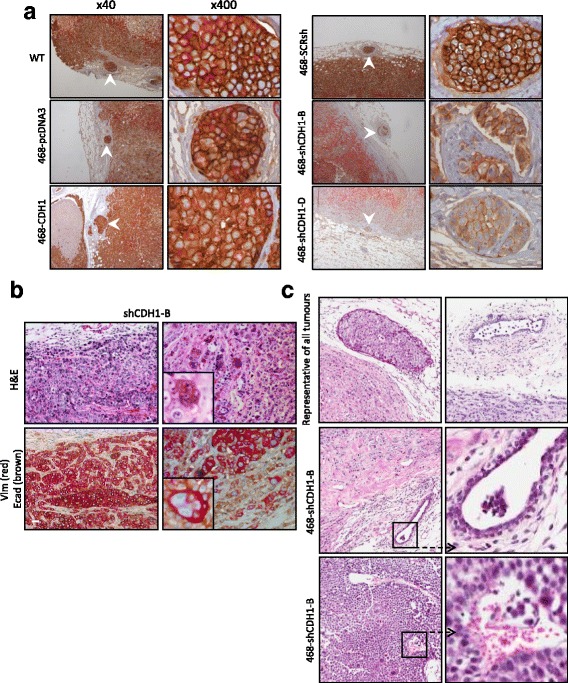
**a**. Dual immunohistochemistry staining (*red* = vimentin, *brown* = E-cadherin) of CDH1-modified cell line xenografts. Original magnification × 40 and × 400. **b** Closer inspection of 468-shCDH1-B (*top*, hematoxylin and eosin [H&E] staining; *bottom*, dual E-cadherin and vimentin staining) with higher-magnification inset. Scale bar = 50 μm. **c** Two types of extratumoral local lymphovascular invasion observed in tumors. Invaded cells presented as tumor emboli composed of well-cohesive tumor cells (*top panel, left*), and more rarely seen invaded cells remained as scattered individual cells (*top row, right*). 468-shCDH1-B tumor emboli were smaller than the rest and composed of loosely adhered cells (*middle row*). Tumor cells were also observed within intratumoral blood vessels of 468-shCDH1-B tumors (*bottom row*). *WT* Wild type

All WT, 468-SCR, and 468-pcDNA3 groups displayed features similar to those shown in Fig. 2. E-cadherin was homogeneously expressed in all groups, except for the 468-shCDH1-B and 468-shCDH1-D tumors, where it was expressed at a less intense level than the control and in a heterogeneous pattern (Fig. 9a, *lower right panel*). Although local lymphovascular invasion (LVI) was seen across all the tumor groups, no clear association was detected between LVI and the E-cadherin status of the primary xenograft (Table 6). Generally, LVI was more apparent in larger tumors, except for the 468-shCDH1-B and 468-shCDH1-D groups, where the LVI was clearly seen even with smaller primary tumors (Fig. 9c).

**Table 6:** Local lymphovascular invasion

Group	Number of mice inoculated	With tumors	With local invasion	Percentage of invasion
468-shSCR	13	13	8	61.5
468-shCDH1-B	13	13	8	61.5
468-shCDH1-D	12	12	11	91.7
468-pcDNA3	12	9	5	41.7
468-CDH1	12	12	11	91.7

Local invasion of the lymphovascular spaces was a common feature in many tumors across all the groups, and no clear association was detected between it and the E-cadherin status of the primary tumors

The majority of tumor emboli seen in vessels were E-cadherin-positive, indicating their epithelial phenotype. Although it is generally accepted that MET occurs to facilitate growth at the secondary site, our observations suggest that it may occur earlier in the bloodstream and further support the requirement of epithelial features, potentially through MET, during metastatic progression.

The presence of multinuclear tumor giant cells is indicative of the highly aggressive nature of the 468-shCDH1-B group (Fig. 9b). These features stained positive for human vimentin, indicating their human origin.

### Trend for association of lung metastases with higher E-cadherin level

Attempts to assess the lung metastasis efficiency of the different MDA-MB-468 cell populations via experimental metastasis were unsuccessful because tail vein injection in SCID mice did not result in any lung metastases in our hands. However, our experiments were compromised by the small number of lung metastases arising from the orthotopic primary xenografts across all groups (Table 4). The incidence of lung metastases, expressed as a percentage of mice in each group with lung metastases, appeared markedly lower in mice carrying 468-shCDH1-B or 468-shCDH1-D xenografts than with the 468-SCR, suggesting that E-cadherin knockdown adversely affected the establishment of secondary colonies; however, this trend observed among the groups was not significant. This assumption was reinforced by the observation that the percentage of mice with lung metastases was slightly higher in the E-cadherin-overexpressing 468-CDH1 group than in the vector control (Table 4). Although the tumors grew at different rates, the mice were harvested as close to the same size as possible (Additional file 2: Figure S2b).

### Correlation of CDH1 protein expression with distant metastasis-free survival in individuals with breast cancer

**Fig. 10:**
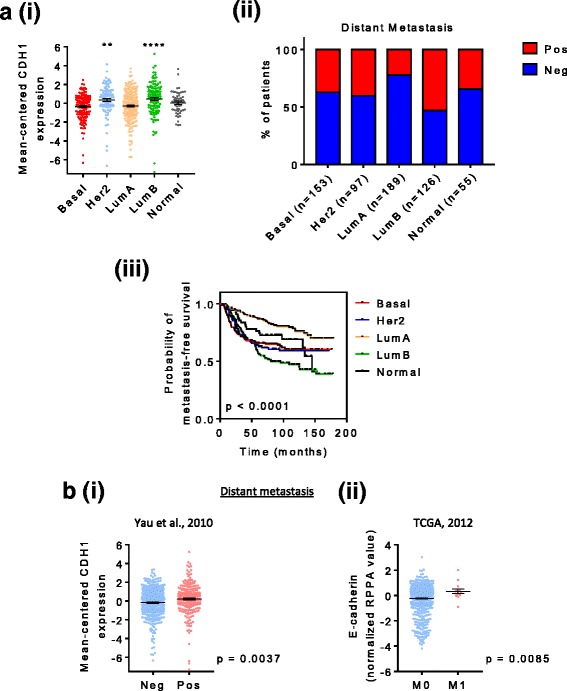
E-cadherin expression correlates with poor clinical outcome for patients with breast cancer. **a** Analysis of clinical data of breast cancers separated according to PAM50 intrinsic subtype from a publicly available microarray study [78]. (*i*) *CDH1* expression. **** *p* > 0.001, ** *p* < 0.01, by one-way analysis of variance versus basal. (*ii*) Percentage of patients positive or negative for distant metastasis. (*iii*) Kaplan-Meier curve depicting the correlation between breast cancer subtype and distant metastasis-free survival. *p* Value was determined by the log-rank (Mantel-Cox) statistical test. **b** Breast cancers that did or did not metastasize to distant sites were analyzed with respect to E-cadherin expression. (*i*) Data from Yau et al. [78], the same data source used for figures shown in (**a**) and (*ii*) [77]. *p* Values were determined by Welch’s *t* test

To determine whether E-cadherin expression predicted distant metastasis formation, an analysis of public breast cancer databases that had outcome data was performed [77, 78]. Patients’ breast tumors were separated according to PAM50 intrinsic subtypes (basal, *HER2*-positive, luminal A, luminal B, or normal), and the mean centered *CDH1* expression was examined (Fig. 10a, *i*). In patients with tumors of the luminal B subtype, E-cadherin expression was expressed the highest (*p* > 0.001 by one-way ANOVA for luminal B versus basal). Of the various tumor subtypes, a greater percentage of samples with distant metastases was observed when they were of the luminal B subtype, and subsequently this tumor type was more likely to present as metastasis within 15 years (Fig. 10a, *ii* and *iii*, respectively) (*p* < 0.0001). When these same data were plotted as the presence or absence of metastasis, *CDH1* expression was a determining factor (Fig. 10b, *i*) (*p* = 0.0037). This was consistent with analysis of The Cancer Genome Atlas breast cancer dataset [77] (Fig. 10b, *ii*) (*p* = 0.0085), in which tumors were separated on the TNM staging scale of M0 being no distant metastasis and M1 indicating tumors that metastasized to distant organs (i.e., beyond the regional LNs).

## Discussion

As previously reported [66], we have shown that the MDA-MB-468 human breast cancer cell line displays significant EMP in vivo, dynamically switching on a mesenchymal phenotype in the primary tumor either at the periphery or in response to HPX (EMT) but exhibiting a predominantly epithelial phenotype in the xenograft center and at the secondary sites in local LNs and the lung. Our in vivo study suggests that in this model, E-cadherin is a major phenotypic driver for metastatic colonization and is lost in EMT sites of the primary tumor, and this was supported by the poorer survival seen in grade III breast cancers expressing high levels of E-cadherin mRNA. The sustained and exaggerated EMT seen after E-cadherin suppression by shRNA in vivo, however, was not readily seen in vitro, suggesting that other factors in the tumor microenvironment are necessary for the full EMT program. Further to this, we have identified that E-cadherin promotes primary tumor growth but may also be important for the formation of lung metastases, thus disputing the previously held notion of E-cadherin as a tumor suppressor [79].

### Evidence challenging the tumor suppressor status of E-cadherin

Evidence for E-cadherin’s role as a tumor suppressor has accumulated, with downregulation of *CDH1* being linked with epithelial tumor progression [79, 80], although its reexpression in tumors is reported to promote metastasis in later stages of tumor progression (reviewed in [14]). Indeed, inactivating mutations of the *CDH1* gene in gastric and lobular breast cancers have defined E-cadherin as a tumor suppressor for these cancer types (reviewed in [81]). However, E-cadherin has an unexpected proneoplastic effect in ovarian cancer [82]. *CDH1* is upregulated in proliferating ovarian cancers, in which its suppression inhibits their proliferation [83, 84] and its proliferation-promoting effects in this context have been shown to occur via mitogen-activated protein kinase kinase/extracellular signal-regulated kinase pathway activation [85]. Perhaps consistent with this, breast cancers expressing higher levels of the epithelium-promoting miR-200 family that represses the *CDH1*-suppressing EMP drivers *ZEB1* and *ZEB2* have poorer outcomes; however, this appeared to be due to mechanisms additional to E-cadherin regulation [86]. Chu et al. [74], who also showed a reduced proliferative state after E-cadherin knockdown in the human breast cancer cell lines MARY-X and SUM149, found that higher levels of E-cadherin in basal breast cancers were associated with poorer outcome, consistent with our observations in luminal B tumors (Fig. 10). *CDH1* and proliferative markers also correlated in a study of endometrial cancer [87] and have been associated with malignant and metastatic potential in bladder and prostate cancer cell lines [88]. We have also shown in the PMC42 human breast cancer model system that cellular proliferation was associated with an epithelial phenotype because the stable knockdown of *ZEB1* in PMC42-ET cells resulted in *CDH1* reexpression along with an increased proliferative rate [38].

### E-cadherin-knockdown cells grew more slowly in vitro and in vivo

In the present study, we have shown that E-cadherin greatly enabled the proliferation of MDA-MB-468 cells in vitro. These in vitro effects were recapitulated in the in vivo setting with dramatically reduced xenograft growth after E-cadherin suppression, with one minor exception: growth of the 468-shCDH1-D cells. These cells plated in vitro exhibited a noticeable reduction in proliferation rate only when plated sparsely (Fig. 4c, *i*). It is reasonable to assume that the growth environment of the tumors formed from these cells would not have been sparse; yet, as shown in Fig. 5a(*ii*), the 468-shCDH1-D tumors grew significantly slower than the control, indicating additional tumor microenvironmental factors at play.

Consistent with our overall findings that E-cadherin enabled cellular proliferation, a subline of the DU145 human prostate cancer cell line with strong E-cadherin expression formed xenograft tumors, whereas a subline of cells with weak E-cadherin expression did not form tumors at all [89]. Moreover, Celià-Terrassa et al. showed that the proliferative, malignant, and metastatic competence of both prostate (PC-3) and bladder (T24-TSU) cell lines required E-cadherin expression, which also coincided with expression of pluripotency genes, and that manipulation of *CDH1* or pluripotency genes reciprocally affected each other [88]. A reduction in tumor growth has also been seen in MFP tumors of E-cadherin-knockdown SUM149 and MARY-X human breast cancer cells, in 4 T1 mouse mammary carcinoma cells, and in SUM149 cells engineered to overexpress *ZEB1*, all of which demonstrated dramatic growth retardation compared with their respective controls [74].

### *HIF-1α* and E-cadherin mechanistic effects on proliferation

Gene expression analysis of the xenograft tumors from Chu et al. [74] revealed that E-cadherin knockdown caused a loss of HPX response genes, including *HIF-1α*, and that normal growth rate was restored in E-cadherin-knockdown SUM149 xenografts by overexpressing *HIF-1α*. In this particular study [74], it was deduced that the E-cadherin shRNA cell lines had a diminished ability to respond to hypoxic stress, and examination of the cells in vitro revealed a reduced capacity for glycolysis, which affected energy availability and growth potential. In the present study, we found that HIF-1α expression was noticeably absent in 468-shCDH1-B tumors (which exhibited the best E-cadherin knockdown), whereas HIF-1α was strongly expressed at the necrotic perimeter in tumors from other cell groups (Fig. 6a, *right panel*). Given that 468-shCDH1-B tumors also grew the slowest in our studies, we considered the possibility that growth of the 468-shCDH1-B tumors was hampered by the inability of these cells to access energy by glycolysis and thus to survive in the hypoxic environment, as shown in E-cadherin knockdown in SUM149 cells [74]. We found, however, that the 468-shCDH1-B cells were not impaired in their ability to upregulate the HPX-inducible gene *CAIX* in hypoxic conditions simulated in vitro (Fig. 6c). It is probable that these tumors did not express HIF-1α because they did not experience HPX, most likely attributable to their better-developed blood vasculature (Fig. 6a, *left panel*) afforded by slower tumor growth.

### Snail1 repressive effects on proliferation

We demonstrated in our previous study that Snail1 and Snail2 were expressed in vimentin-positive zones within the MDA-MB-468 xenografts [66]. These act directly on the cell cycle to downregulate cellular proliferation [90–92]. The 468-shCDH1-B tumors that were slowest-growing also homogeneously expressed vimentin. These tumors also showed upregulated *SNA1* (Fig. 8), which may have led to the observed slowed growth of these tumors (Fig. 5a, *ii*; Additional file 2: Figure S2a). Loss of E-cadherin at the cell membrane may have fueled *SNA1* transcription via freed β-catenin acting in the nucleus [93]. ESR1 was downregulated in the 468-shCDH1-B tumors, a transcriptional target of *SNA1* in EMT [76]. This provides support for the hypothesis that, in addition to upregulated *SNA1* mRNA in the 468-shCDH1-B tumors, this resulted in upregulated Snail1 protein, which was transcriptionally active.

### E-cadherin loss does not always result in reduced cellular proliferation

Although we have reviewed several lines of evidence to show that E-cadherin expression may provide a certain growth advantage in vivo, both in the onset of primary tumors and in their metastases, contradictory data suggest that loss of E-cadherin in some cell contexts does not reduce proliferation, and this is an important caveat. Indeed, proliferation is not always downregulated in EMP [94], and the EMP regulator and *CDH1* suppressor *ZEB1* plays a pro-proliferative role in certain contexts [95, 96]. The lack of EMP in lobular breast cancer, which is defined by loss of E-cadherin, suggests that lobular carcinoma cells have constraints that avoid any manifestation of EMP [67], and their lack of E-cadherin does not appear to compromise their ability to grow, perhaps due to some genomic override. In mammary systems, the relationship between EMP and stemness was accompanied by reduced proliferation in some studies [9, 43]; however, EMP induced by ectopic expression of *FOXC2* in EpRas cells did not affect either their proliferation rate in vitro or the growth kinetics of the resulting primary tumors in nude mice [29]. Ongoing stemness after MET has been suggested in studies examining *PRRX1*, a gene mediating EMT in migrating and invading cancer cells; downregulation of *PRRX1* enables epithelial reversion, maintenance of stemness, and metastasis formation [50]. It is important to appreciate that the positive relationship between E-cadherin and proliferation described here, and as seen in several other systems, is not universal; however, the explanation for this remains unclear.

### E-cadherin-knockdown cells displayed a pronounced EMT within the tumor microenvironment

The extent of in vivo vimentin expression across the various tumor groups strongly correlated with E-cadherin status (Fig. 7a). This was best observed in 468-shCDH1-B tumors, which displayed the best E-cadherin knockdown (Fig. 3B), and also displayed the highest and most homogeneous expression of vimentin protein (Fig. 7a) and mRNA (Fig. 8). Upregulated *VIM* expression was not observed in the 468-shCDH1-B or 468-shCDH1-D cells when plated in 2D or 3D (mRNA; Fig. 3c, *ii*, and Figs. 7c and Fig. 8, respectively), nor did the altered *SNA1*, *ESR1, INHBA*, and *LAMC2* expression (from control) observed in tumors (Fig. 8) correlate in in vitro culture of the corresponding cells (Fig. 8). Induction of EMT by EGF did occur more easily (Fig. 3C), however. These results are similar to the findings of Chen et al., who showed that stable loss of E-cadherin in MCF10A cells did not drive EMT [97] and that loss of E-cadherin was not necessary or sufficient to induce EMT in cultured breast cells [98], as opposed to studies where E-cadherin loss did lead to EMT in vitro [71, 99]. It is likely that the in vivo EMT in the present study was influenced by murine stromal factors or biophysical influences. These stromal factors may also have induced an EMT at the tumor-stroma border in 468-WT tumors (Fig. 1a). One plausible host stromal candidate could include mast cell-derived interleukin-6, which we have previously implicated in a positive feedback cycle between tumor cells and host myofibroblasts in MDA-MB-468 xenografts [100], although other soluble factors, microRNAs, and exosomes have been found to be released from tumor-activated stromal cells to promote EMT (reviewed in [101]). It is also possible that the duration of culture of these cells in the mouse (up to 154 weeks) led to different outcomes than seen in the relatively shorter time frames used in vitro.

### E-cadherin expression in metastases: importance of MET in secondary tumor formation

WT MDA-MB-468 tumor cells that were found in the lung as micrometastases expressed E-cadherin, whereas no vimentin-positive cells were observed (Fig. 2b). Cells from the same primary tumor that invaded into the axillary LNs also demonstrated E-cadherin expression, with the E-cadherin signal in these cells even stronger than that of the primary tumor (Fig. 2c). E-cadherin expression may be advantageous for the formation of these lung metastases because we observed a trend toward a higher number of micrometastases in this organ in the mice carrying xenografts with forced E-cadherin (468-CDH1) than in their controls, as well as a reduced number when E-cadherin was knocked down despite the considerably longer tumor exposure time (Table 4). The sh-CDH1 xenografts grew slower, so the potential to seed metastases may have been reduced, and the lung metastases may have also grown slower. Kowalski et al. [102] reported results showing distant metastases expressing an E-cadherin signal equal to or stronger than that of the respective primary tumors from which they originated. They saw all metastatic tumors of invasive ductal carcinoma reexpressing E-cadherin, regardless of the E-cadherin status of the primary tumors. In other studies, Saha et al. [103] showed reexpression of E-cadherin in bone metastases that originated from E-cadherin-negative, poorly differentiated primary breast carcinoma, and Chao et al. [46] reported the reexpression of E-cadherin at distant metastases arising from E-cadherin-low or E-cadherin-negative primary tumors. They reported strong E-cadherin expression in more than 50% of liver, brain, and lung metastasis originating from infiltrating ductal carcinoma of the breast, as well as in lung metastases from E-cadherin-negative MDA-MB-231 primary xenografts. They suggested that the reexpression of E-cadherin in metastases was influenced by the microenvironment of the metastatic site. To investigate their hypothesis, they demonstrated that the E-cadherin-negative MDA-MB-231 cells expressed E-cadherin when cocultured with hepatocytes. Similarly, activation of fibroblasts at the metastatic niche is mediated by *AXL*, expressed by mesenchymal circulating cancer cells homing to the niche, which has been shown to be necessary for metastatic colonization and MET [104]. Gao et al. showed that bone marrow-derived myeloid precursor cells produced versican that could neutralize transforming growth factor β1 in the lung, promoting MET and metastatic competence in MDA-MB-231 cells [105]. MET may also occur in circulating tumor cells (CTCs) shed from the primary tumor, as has been shown in small cell lung cancer, where it has been postulated that the large CTC clusters that form may do so in response to the selection pressure of first-line chemotherapy [106]. Assessment of CTCs in a separate study of MDA-MB-468 xenografts showed significantly increased expression of both mesenchymal (*SNAIL1*, Notch homolog 1, translocation-associated [*Drosophila*] [*NOTCH1*], *SERPINE1*, insulin-like growth factor 1 receptor, Ras-related protein R-Ras, neurophilin 1, *INHBA*), and epithelial (B-lymphocyte antigen *CD20, CDH1*, bone morphogenetic protein 7, claudin 3) markers (Tachtsidis, Li et al., unpublished data). *VIM* was increased, but not significantly. These findings, combined with those of the present study, support the importance of epithelial phenotype/MET tumor cell survival in the bloodstream and in the formation of secondary tumors and a central role for E-cadherin in this mechanism [107, 108].

### Features of aggressiveness in 468-shCDH1-B tumors

Consistent with their more invasive status as established by the Transwell migration assays (Fig. 4a, *ii*), 468-shCDH1-B displayed classical “high-grade” tumor aggressiveness features such as multinucleated giant cells (Fig. 9b) and an Indian file histology characteristic of lobular breast cancers, which are defined by a lack of E-cadherin. The presence of such morphologies in invasive breast carcinoma has been reported, although the origin of those cells has been a subject of controversy [109–111]. Factor et al. suggested that giant cells are histiocytic descendants [109], whereas Kobayashi et al. proposed that they originated from the tumor cells through abnormal cell division [110]. Another study suggested that tumor giant cells are generated through the fusion of mononuclear stromal cells other than histiocytes [111]. Given that these cells expressed human vimentin, it appeared that these giant cells were of tumor origin. MDA-MB-468-shCDH1-B tumors grew more slowly than controls (Fig. 5a, *ii*), and thus, combined with the expression of these aggressive phenotypic features, these findings challenge the generally accepted view of “the higher the grade, the more aggressive and fast-growing the cancer.”

### E-cadherin expression determines distant metastasis formation

In our study, we have shown that the E-cadherin-knockdown cell lines displayed a trend toward fewer lung metastases (Table 4), indicating that E-cadherin is important for the formation of these secondary tumors. A previous study looking at E-cadherin expression in tumors of all types and patient survival found that E-cadherin did not have predictive value [86], whereas another study showed that E-cadherin expression (in basal tumors specifically) was associated with a poor prognosis [74]. The clinical data shown in Fig. 10 are consistent with this latter finding and demonstrate that E-cadherin expression is a feature of tumors that metastasize to distant sites, concordant with the results of another study in which it was shown that luminal B breast cancers are associated with a poorer prognosis [112]. In this particular study, they showed that this breast tumor subtype was also more proliferative, displaying a higher Ki-67 score. This is consistent with our finding that E-cadherin promotes a proliferative phenotype, as shown in Figs. 4 and 5. Taken together, E-cadherin expression in breast cancer has significant implications for patient outcome.

## Conclusions

We have shown that E-cadherin expression sustains proliferation in MDA-MB-468 breast cancer cells in culture and within the growing tumor at both orthotopic and metastatic sites in the SCID mouse. We have also demonstrated that EMP is influenced by the tumor microenvironment and is stimulated by HPX and stromal interactions. We have shown that predominantly epithelial features are seen in tumor-associated LVI, proximal vascular emboli, and local LNs, as well as in lung metastases, and these are consistent with our independent studies of CTCs in this model. Our review of the clinical data with respect to E-cadherin expression in breast tumors support that E-cadherin expression positively predicts the formation of metastases. These findings indicate the requirement of a reappraisal of the precise role of E-cadherin in predicting primary tumor progression and metastatic risk.

## Additional files

Additional file 1: Figure S1. **a** Further examples of MDA-MB-468 cell xenograft images shown in Fig. 1b. (PDF 203 kb)

Additional file 2: Figure S2. **a** Comparison of individual tumor growth plots for 468-shCDH1-B and 468-shCDH1-D tumors to day of tumor harvest. **b** Tumor volumes at time mice were killed did not differ greatly. (PDF 106 kb)

Additional file 3: Figure S3. Ki-67 immunostaining of the various E-cadherin-modified murine tumors, showing no difference in number of positive nuclei.tr (PDF 283 kb)

## Abbreviations

AEC: Animal ethics committee
ANOVA: Analysis of variance
BSA: Bovine serum albumin
*CAIX*: Carbonic anhydrase 9
*CDH1*: E-cadherin gene
CTC: Circulating tumor cell
DAB: 3,3′-Diaminobenzidine
DAPI: 4′,6-diamidino-2-phenylindole
DPX: Dibutylphthalate polystyrene xylene
EGF: Epidermal growth factor
EMP: Epithelial-mesenchymal plasticity
EMT: Epithelial-to-mesenchymal transition
ER: Estrogen receptor
*ESR1*: Estrogen receptor alpha
*FOXC2*: Forkhead box protein C2
G418: Geneticin
GFP: Green fluorescent protein
H&E: Hematoxylin and eosin
HIF: Hypoxia-inducible factor
HPX: Hypoxia
IgG: Immunoglobulin G
IHC: Immunohistochemistry
INHBA: Inhibin, beta A
LN: Lymph node
LVI: Lymphovascular invasion
MET: Mesenchymal-to-epithelial transition
MFP: Mammary fat pad
miR: MicroRNA
mRNA: Messenger RNA
NOTCH1: Notch homolog 1, translocation-associated (*Drosophila*)
OD: Optical density
qPCR: Quantitative polymerase chain reaction
SCID: Severe combined immunodeficiency
SCR: Scrambled
*SERPINE1*: Serpin family E member 1
shRNA: Short hairpin RNA
SNAI1: Snail family transcriptional repressor 1
*SNAI2*: Snail family transcriptional repressor 2
SRB: Sulforhodamine B
TCA: Trichloroacetic acid
TCGA: The Cancer Genome Atlas
UTR: Untranslated region
WT: Wild type
*ZEB1*: Zinc finger E-box-binding homeobox 1
*ZEB2*: Zinc finger E-box-binding homeobox 2
ΔCT: Cycle threshold change
