# Supplementary figures and images for: Epithelial requirement for in vitro proliferation and xenograft growth and metastasis of MDA-MB-468 human breast cancer cells: oncogenic rather than tumor-suppressive role of E-cadherin
Source: Breast Cancer Res. 2017 Jul 27;19:86. doi: 10.1186/s13058-017-0880-z (PMC5530912; doi:10.1186/s13058-017-0880-z)

A

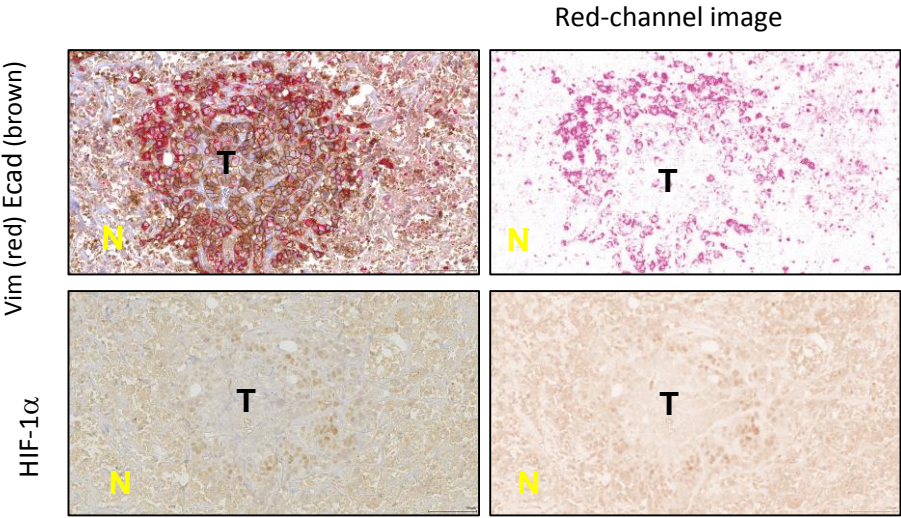

Supplement: Supplementary file 1 — a Further examples of MDA-MB-468 cell xenograft images shown in Fig. 1b. (PDF 203 kb) [file 13058_2017_880_MOESM1_ESM.pdf]

A

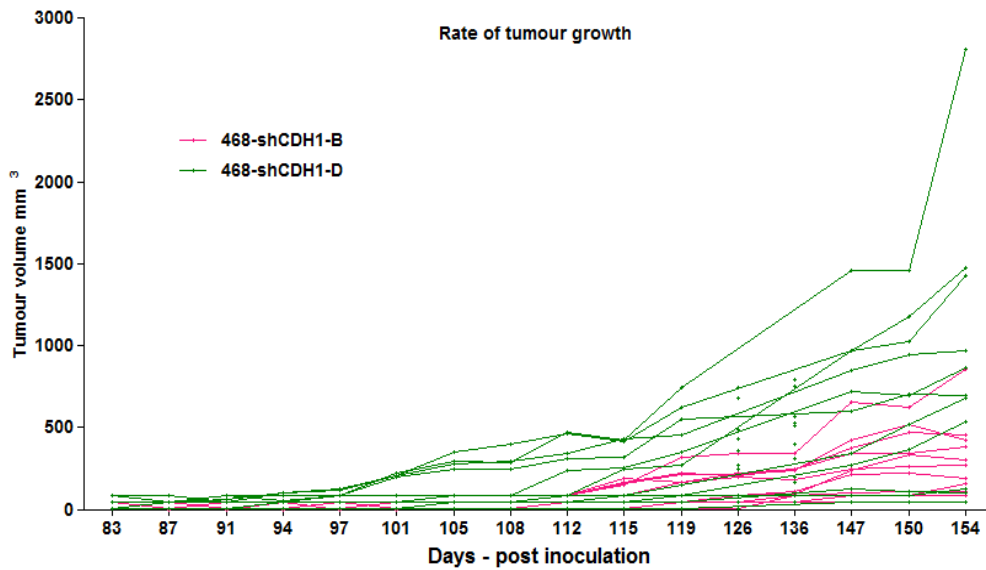

B

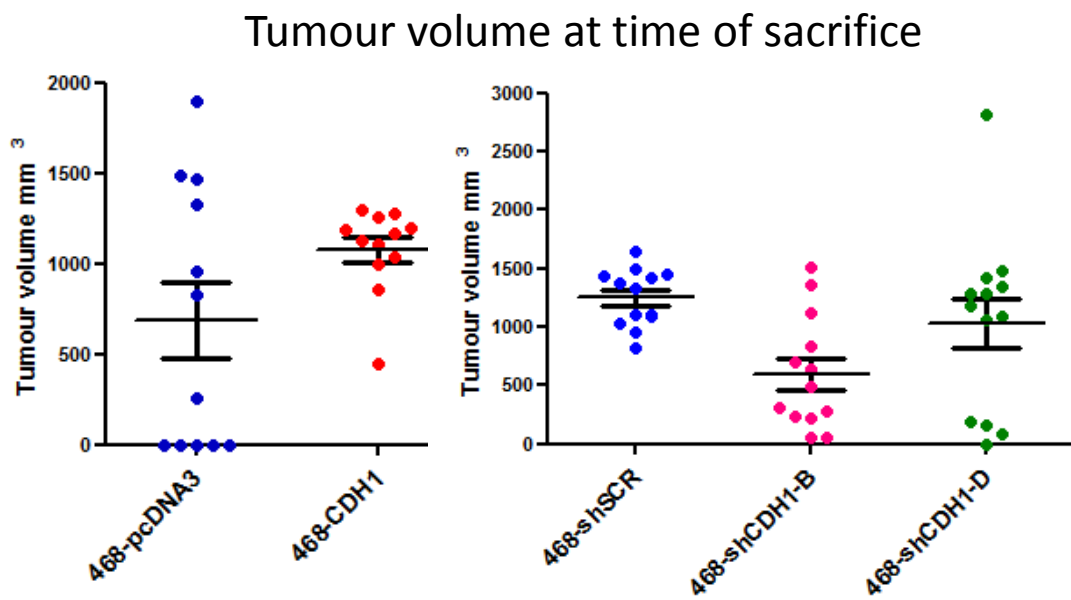

Supplement: Supplementary file 2 — a Comparison of individual tumor growth plots for 468-shCDH1-B and 468-shCDH1-D tumors to day of tumor harvest. b Tumor volumes at time mice were killed did not differ greatly. (PDF 106 kb) [file 13058_2017_880_MOESM2_ESM.pdf]

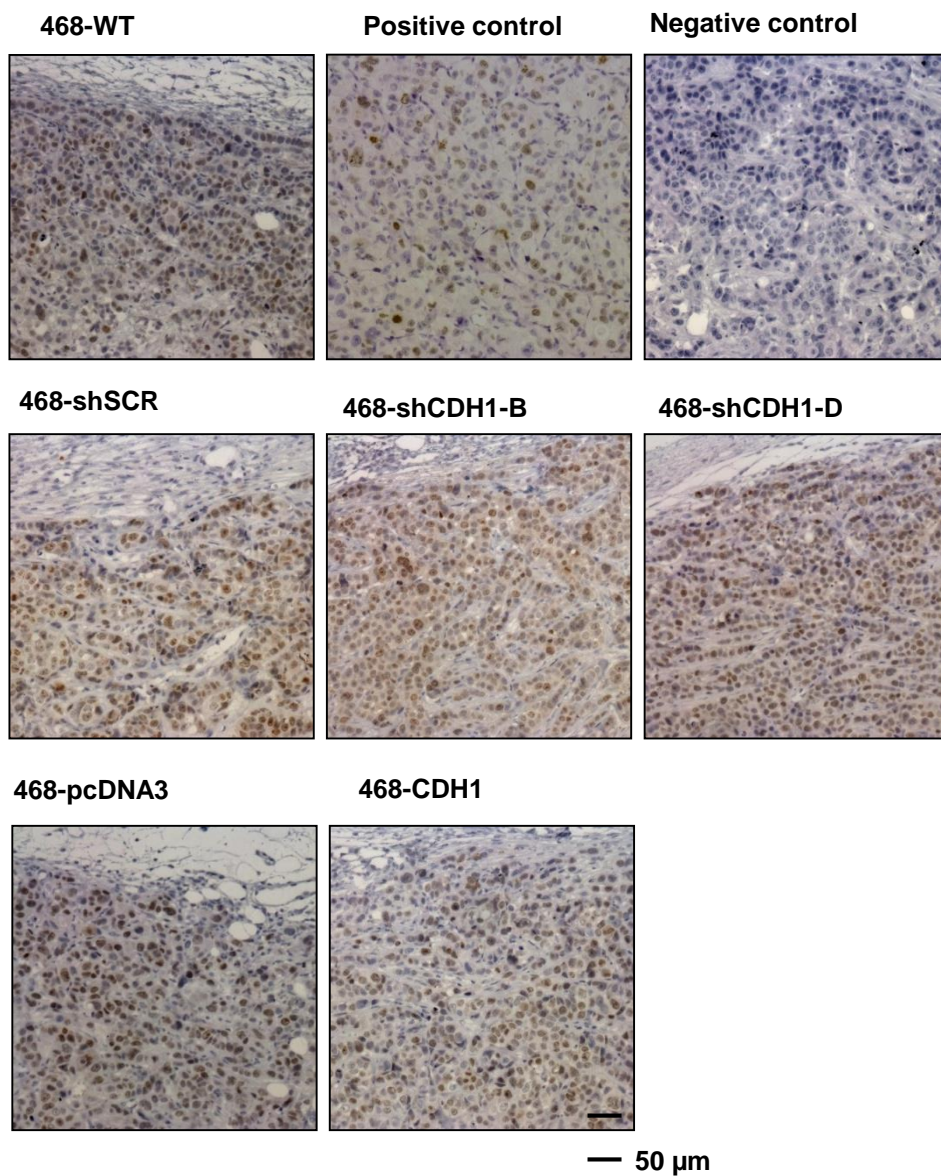

Supplementary figure 3

Supplement: Supplementary file 3 — Ki-67 immunostaining of the various E-cadherin-modified murine tumors, showing no difference in number of positive nuclei.tr (PDF 283 kb) [file 13058_2017_880_MOESM3_ESM.pdf]
